# Rare retro‐patellar multiple osteochondromas in patellar tendon: A case report

**DOI:** 10.1002/ccr3.7751

**Published:** 2023-07-30

**Authors:** Hosein Pirmohamadi, Mohsen Rahimi, Mohammad Kazem Emami Meibodi, Mohsen Akbaribazm

**Affiliations:** ^1^ Trauma Research Center Baqiyatallah University of Medical Sciences Tehran Iran; ^2^ Health Research Center, Life Style Institute Baqiyatallah University of Medical Sciences Tehran Iran; ^3^ Department of Basic Medical Sciences Khoy University of Medical Sciences Khoy Iran

**Keywords:** exostosis, knee joint, osteochondromas, patellar ligament, radiography

## Abstract

Osteochondromas (OCs) are developmental anomalies that originate from the periosteum and typically form during enchondral ossification near the joints. Retro‐patellar OC caused by exostosis forms in various intracapsular, intra‐tendon, and joint‐adjacent positions within the knee joint. In this case, a 19‐year‐old male presented with swelling and a mass in his left knee, which raised suspicion of bone tumors. After evaluating x‐ray images and conducting histopathological examinations, the diagnosis was confirmed as retro‐patellar OC.

## Graphical abstract

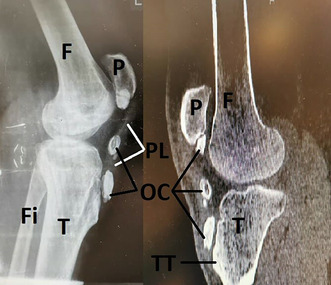

## 1 INTRODUCTION

Osteochondromas (OCs) are benign tumors that account for 20%–50% of all benign and 10% of all bone tumors. The incidence rate of OC is twice as high in males as it is in females, with the peak occurrence usually observed during the second decade of life.^1^ Multiple hereditary exostosis is an autosomal dominant inheritance that results in the formation of isolated lesions or multiple exostoses during the development of bones through the process of enchondral ossification in long bones.^2^ Additionally, OC has been linked to mutations in some tumor suppressor genes, including exostosin‐1 (EXT1) and exostosin‐2 (EXT2) genes located at 8q24 and 11p11‐p12, respectively. OC originates from the periosteum and occurs in the active parts of bones, including the metaphysis of long bones and the cartilage at their ends. Studies suggest that OC can also result from radiation‐induced injury, hematopoietic stem cell transplantation, and surgery.^3^

Osteocartilaginous exostosis is typically detected in childhood as a palpable mass, often accompanied by chronic pain and sometimes edema.^4^ One of its common manifestations is the presence of misplaced bone masses within the joint capsule, sometimes with cartilaginous coating visible on radiographic images. Depending on the size, location, and stage of tumorigenesis, various preoperative diagnostic tools can be used to diagnose OC masses, including simple radiographs, magnetic resonance imaging (MRI), computed tomography (CT), and bone scintigraphy. The hip and knee joints are the most commonly affected joints. While patellar OCs are rare, they typically affect the patellar bursa. For example, Moraes et al. (2014) reported a case of painless, 8 × 6 × 3 cm patellar OC located anterior to the patella in a 60‐year‐old man with no limitation of flexion‐extension in the knee joint.^5^ In the present case, the rare retro‐patellar OC was observed in the area of the patellar ligament.

## 2 CASE REPORT

**FIGURE 1:**
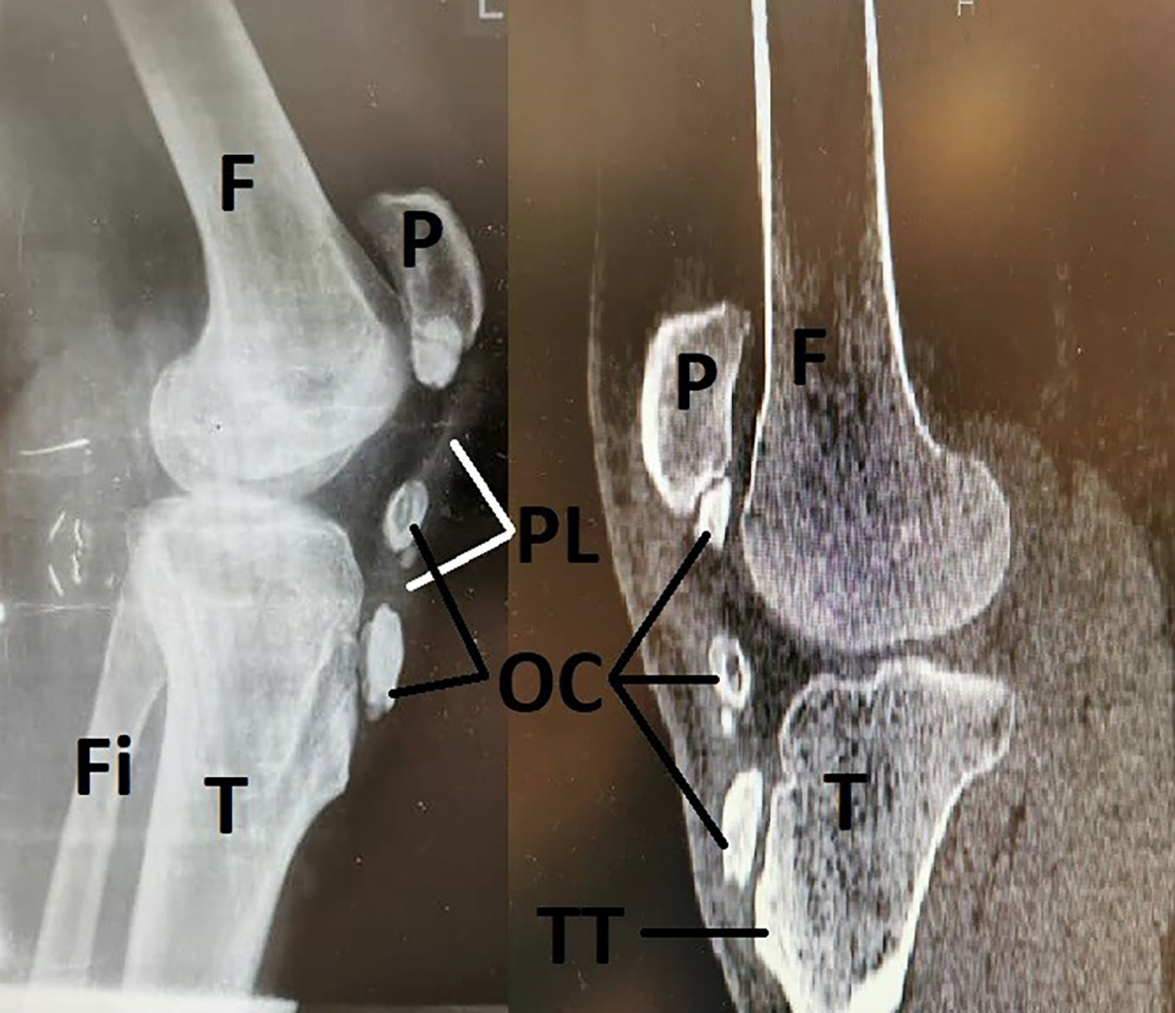
x‐Ray image of the left knee joint. Multiple OC within the patellar tendon. Bone wear (asterisk) is evident at the femoral condyle and tendon attachment to the tibial tuberosity. F, femur; Fi, fibula; OC, OC masses; P, patella; PL, patellar ligament; T, tibia; TT, tibial tuberosity.

A 19‐year‐old male presented to Baghiyyatollah al‐Azam military hospital on December 13th, 2022, with chronic pain and a swelling, hard, and immobile mass in the antero‐inferior region of his left knee. The mass had grown over a year. After examination, with the help of lateral radiography of the left knee (as shown in Figure 1), irregularities were observed in the inferior patella joint surface and multiple exostosis in the patellar tendon region. Tumor masses were palpable in the left patellar tendon, and there was a progressive limitation of movement in flexion and extension of the knee joint. The tumor lesion had sharp borders and a hyper‐dense appearance, and there were no signs of fracture or dislocation observed in the tibiofemoral and patellofemoral joints.

**FIGURE 2:**
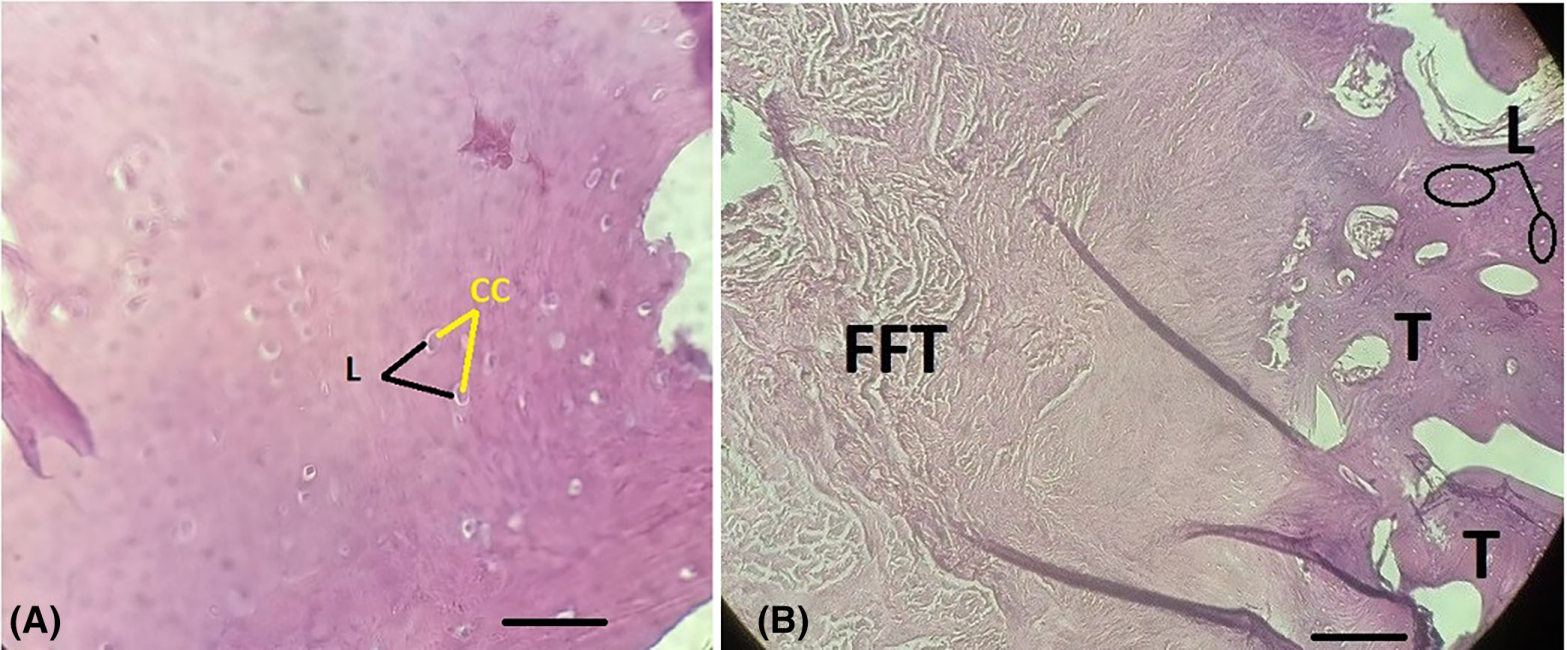
Histopathology of OC masses. Proliferated chondroid and osteoid tissue which covered with FFT and thick hyaline cartilage. Lacunae containing isogenous chondrocytes (CC) buried in the forming trabeculae with calcified region. FFT: fibro‐fatty tissue, T: bone trabeculae (bone matrix‐calcified region), L: osteocytic lacunae. [(A) H&E staining × 100, scale bar = 200 μm and (B) H&E staining ×400, scale bar = 50 μm].

Following the spinal anesthesia, an anterior skin incision was made and the patellar tendon was opened using a mid‐patellar approach. In addition, masses were excised using radical resection. After performing a proximal osteotomy of the left tibia for better access, three irregular bone masses with a total size of 7 × 5 × 1 cm were removed and sent for histopathological examination. Sutures were removed 2 weeks after surgery then rehabilitation was started to improve flexion and extension. After 2 weeks, the patient's knee joint was immobilized and physiotherapy was initiated. After 2 months, there were no limitations in the patient's range of motion. Histopathological evaluation revealed the presence of well‐differentiated trabeculae‐spongy bone tissue, containing proliferated chondroid and osteoid areas in hypodense lacunae, which were covered by fibro‐fatty tissue and thick hyaline cartilage (as shown in Figure 2). This evaluation also confirmed multiple exostoses in the patellar tendon. Differential diagnosis included bizarre parosteal osteochondromatous proliferation (Nora's Lesion) and florid reactive periostitis. At the end of the 3‐month follow‐up, the patient had no movement disorders related to flexion and extension of the knee joint, pain, or tumor lesions.

## 3 DISCUSSION

OC is a benign tumor of bone tissue that typically develops during the growth of long bones through endochondral ossification. OC is often associated with hereditary multiple exostoses and occurs in the areas around the tibiofemoral and patellofemoral joints.^6^ While this lesion is rare in the patellar ligament, it can cause knee joint stiffness (joint locking), dysfunction of the quadriceps femoris muscle tendon, loss of knee joint extension, edema, chronic pain, ossification of the ligament, formation of fibro‐fatty tissue, degenerative arthritis, and neurovascular compression.^7^

In this case, the patient exhibited common symptoms of OC, including edema, chronic pain, stiffness in the knee joint, and palpation of solid masses in the patellar ligament. These symptoms may be due to the inflammatory processes involved in the degeneration of dense connective tissue around the mass of exostoses in the lacunae of the patellar ligament. Studies indicate that OC is associated with a good prognosis and low risk of metastasis. However, due to the disturbance in the anatomical alignment of the knee joint, masses are typically removed by radical resection, and the patellar ligament is fixed and stabilized at the connection to the tibial tuberosity.^5^ Pandian et al. (2016) reported a case in which a 22‐year‐old man had bilateral OC masses in the patella and patellar ligament, causing severe arthritis, chronic pain, edema, and impaired flexion‐extension knee movements.^8^ In 15% of cases, the mass is surrounded by a calcified cartilage cap around the bony lacuna, and its thickness may even exceed 15 mm. Tumor lesions are also surrounded by 1–10 cm of cartilaginous cap, which, in the deeper parts, contains trabeculae of spongy bone tissue and osteocyte lacunae secreting bone calcification matrix.^4^ Therefore, histopathological evaluations of OC masses typically reveal three layers of spongy (trabeculae) bone forming region, thick hyaline cartilage, and perichondrium containing fibro‐fatty tissue, from the inside to the outside.

In this case, the OC masses of thick hyaline cartilage have chondrocyte lacunae with isogenic accumulations, surrounded by spongy bone tissue containing calcified trabeculae and perichondrium containing fibro‐fatty tissue. The incidence of OC ranges from 0.9 to 1.4% per 100,000, making it rare in the patellar ligament. Without intervention, valgus deformity in the ankle, knee, and pelvic joints is expected in the third decade of life. However, vascular compression and/or nerve compression along with acute pain, extensive edema, and tissue degeneration are observed in OC of the hip (sacroiliac‐iliofemoral) and shoulder (glenohumeral) joints.^9^, ^10^ Malignant transformation (secondary OC) is more frequent in multiple hereditary exostoses than solitary OC. Low‐grade malignancy with loss of cartilage architecture, mitotic activity, presence of cell atypia, and necrosis may indicate secondary malignant transformation.^11^

## 4 CONCLUSION

OC is a benign tumor that develops in the articular‐metaphyseal areas of long bones and rarely causes intra‐articular and intraligamentous lesions. When it occurs in the patellofemoral joint, it is a rare condition that usually results in limited flexion/extension movements of the knee joint and stiffness, as well as chronic pain in the affected area. Treatment options for patellofemoral joint OC include radical resection of the mass.

## AUTHOR CONTRIBUTIONS

**Hossein Pirmohamadi:** Conceptualization; supervision; writing – review and editing. **Mohsen Rahimi:** Supervision; writing – review and editing. **Mohammad Kazem Emami Meibodi:** Supervision; writing – review and editing. **Mohsen Akbaribazm:** Conceptualization; supervision; writing – original draft; writing – review and editing.

## FUNDING INFORMATION

This case report study was not financially supported by any organization.

## CONFLICT OF INTEREST STATEMENT

The authors declare that there is no conflict of interest.

## CONSENT

Written informed consent was obtained from the patient to publish this report in accordance with the journal's patient consent policy.

## Data Availability

All data associated with the article are available if required.
